# Towards Improved Quality of GPCR Models by Usage of Multiple Templates and Profile-Profile Comparison

**DOI:** 10.1371/journal.pone.0056742

**Published:** 2013-02-28

**Authors:** Dorota Latek, Pawel Pasznik, Teresa Carlomagno, Slawomir Filipek

**Affiliations:** 1 International Institute of Molecular and Cell Biology, Warsaw, Poland; 2 EMBL, Structural and Computational Biology Unit, Heidelberg, Germany; 3 Faculty of Chemistry, University of Warsaw, Warsaw, Poland; Aberystwyth University, United Kingdom

## Abstract

G-protein coupled receptors (GPCRs) are targets of nearly one third of the drugs at the current pharmaceutical market. Despite their importance in many cellular processes the crystal structures are available for less than 20 unique GPCRs of the Rhodopsin-like class. Fortunately, even though involved in different signaling cascades, this large group of membrane proteins has preserved a uniform structure comprising seven transmembrane helices that allows quite reliable comparative modeling. Nevertheless, low sequence similarity between the GPCR family members is still a serious obstacle not only in template selection but also in providing theoretical models of acceptable quality. An additional level of difficulty is the prediction of kinks and bulges in transmembrane helices. Usage of multiple templates and generation of alignments based on sequence profiles may increase the rate of success in difficult cases of comparative modeling in which the sequence similarity between GPCRs is exceptionally low. Here, we present GPCRM, a novel method for fast and accurate generation of GPCR models using averaging of multiple template structures and profile-profile comparison. In particular, GPCRM is the first GPCR structure predictor incorporating two distinct loop modeling techniques: Modeller and Rosetta together with the filtering of models based on the Z-coordinate. We tested our approach on all unique GPCR structures determined to date and report its performance in comparison with other computational methods targeting the Rhodopsin-like class. We also provide a database of precomputed GPCR models of the human receptors from that class.

**Availability:**

GPCRM server and database: http://gpcrm.biomodellab.eu

## Introduction

G-protein coupled receptors form a large membrane protein family consisting of five classes: Rhodopsin-like, Glutamate, Adhesion, Secretin and Taste/frizzled-like receptors [1]. So far, only receptors belonging to the Rhodopsin-like class were studied by crystallography, which provided 3D structures of their apo forms [2] as well as of their complexes with small ligands [3] or other protein domains e.g. a G protein domain [4]. Determination of the first GPCR structure–that of rhodopsin in 2000 [5] was followed by studies on the beta-1 adrenergic receptor (β1AR) in 2007 [6]. Recently, the use of lysozyme [6] or nanobody molecules [7] to stabilize GPCRs accelerated the process of structure determination and in 2012 the number of X-ray PDB entries related to GPCRs reached 130 with 14 unique receptor structures. Despite the recent progress in experimental methods for studying GPCRs there is still a large number of those receptors, involved in endocrine, metabolic or mental processes, for which 3D structures have not yet been solved. To meet the expectations not only of the research community but also of the pharmaceutical industry, as approximately one third of currently available drugs target GPCRs, we propose GPCRM - a new comparative modeling method for fast and accurate structure prediction of GPCRs belonging to the Rhodopsin-like class.

**Figure 1:**
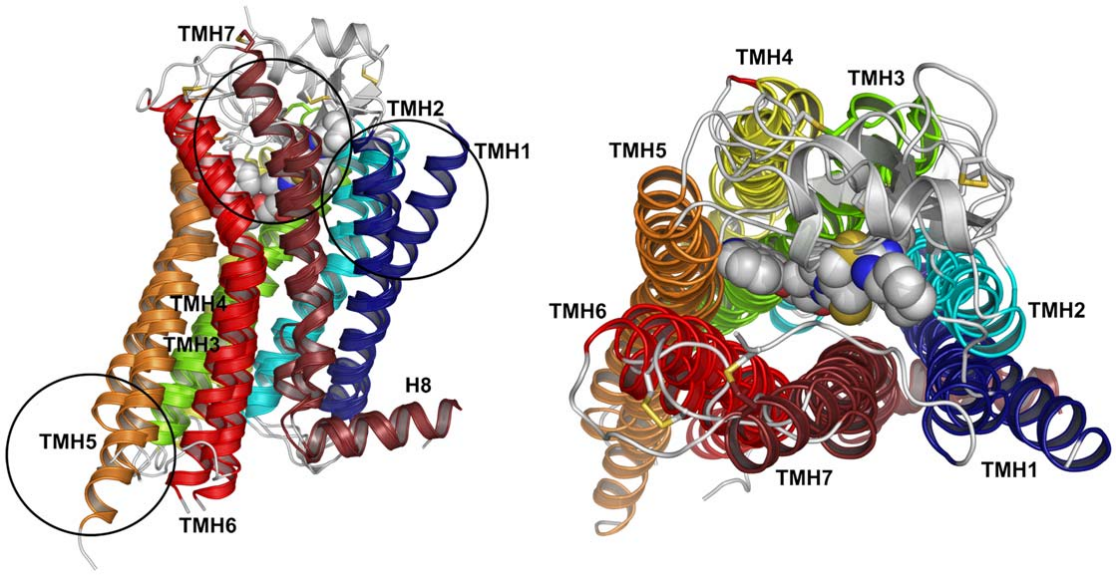
A scheme of 7TMH fold of Rhodopsin-like class of GPCRs. Here, we superposed crystal structures of three GPCRs of varied loop conformations: chemokine CXCR4 (PDB id: 3ODU), adrenergic β2AR (2RH1) and adenosine A2AR receptors (2YDV). Except for variety of loop conformations, GPCR structures differ by kinks in TM helices, e.g., in TMH1 (dark blue) and TMH5 (orange), and the length of TM helices, e.g., of TMH7 (dark red).

Although all GPCRs are believed to share the same 7 transmembrane helices fold (7TMH) they significantly differ in loop conformations, presence of helical kinks or other deformations of TM helices represented by bulges (see Figure 1). Even if structural differences between two GPCRs are negligible as between β1AR and β2AR receptors a few differently oriented amino acids side chains might completely change the binding mode of endogenous or exogenous ligands. For those reasons structure prediction of GPCRs is considered to be a challenge. In general, computational methods based on sequence homology performed much better in GPCR structure prediction than the *de novo* methods, as it was proved by the last GPCRDock 2010 competition [8]. In general, due to the relatively low number of membrane proteins in PDB, their *de novo* structure prediction is less accurate and thus less common than in the case of globular proteins. Notable exceptions are two recently developed methods: Rosetta-membrane [9] and FILM3 [10] (see Table 1). Another interesting example is the protein folding *de novo* based on evolutionary-based constraints (EVfold), recently tested on membrane proteins [11].

**Table 1:** Web services and stand-alone academic applications targeting structure prediction of membrane proteins.

Name	Target proteins	Description	Reference
GPCRM	GPCRs	Comparative modeling by Modeller & Rosetta & multiple template approach & profile-profile alignment	The current publication.
GPCR-ITASSER	GPCRs	Comparative modeling by I-TASSER threading method	[12]
GPCR-ModSim	GPCRs	Comparative modeling by Modeller	[14]
SSFE	GPCRs	Comparative modeling by Modeller & multiple template approach	[39]
Rosetta-membrane	All membrane	Fragment-assembly & membrane proteins-based statistical potentials	[9]
FILM3	All membrane	Fragment-assembly based on the Fragfold method	[10]
ModWeb/ModBase	All membrane & globular	Comparative modeling by Modeller	[60]
Medeller	All membrane	Membrane-specific alignment generation & fragment-based loop modeling	[16]
EVfold/EVfold_membrane	All membrane & globular	Evolutionary-based constraints used in protein folding	[11]

Besides the *de novo* modeling also the homology-based methods which target membrane proteins are still at the stage of development. As the main interest was focused on GPCRs due to their importance in various metabolic pathways some homology-based methods were developed specifically for this family (see Table 1 for details), e.g., academic web services such as: GPCR-I-TASSER [12], Modeller-based services: SSFE [13] and GPCR-ModSim [14] and finally a commercial GPCR helix manipulator in Maestro (Schrödinger, LLC. New York. 2012). Homology models of GPCRs can also serve as a starting point in further *de novo* modeling performed, for example, by manipulation of the orientation angles of transmembrane helices, like in the GEnSeMBLE method [15], yet with moderate success [8]. Notable examples of comparative modeling methods not only for GPCRs but for all membrane proteins are: commercial Yasara (Yasara Bioscience, Vienna 2012) and academic Medeller [16] (see Table 1).

A well-established pipeline for comparative modeling of GPCRs begins with detection of close homologs with solved 3D structures, followed by alignment generation (the core idea of Medeller [16]), model building (commonly performed by Modeller [17]), loop refinement (performed e.g. by SuperLooper ([18]) and a final, though in many cases not necessary [8], step of molecular dynamics relaxation simulation in a membranous environment (a claimed functionality of GPCR-ModSim [14]). Although GPCRM is not the first approach to comparative modeling of GPCRs, it is the first method which integrates in a single pipeline various programs which currently perform best in all the modeling steps mentioned above. GPCRM uses novel features such as a profile-profile comparison and model building based on averaged multiple templates combined with implicit information about the membrane location. The concept of profile-profile comparison is well-established in the field of bioinformatics and was used successfully in detecting distant sequence homology [19]–[22] and producing more accurate sequence alignments [23]–[27]. Surprisingly, in recent studies involving GPCRs, the usage of sequence profiles has been mostly limited to classification purposes [28] and detection of binding sites [29] with very few examples of implementation in structure modeling [30] or improvement of sequence alignment [8].

**Figure 2:**
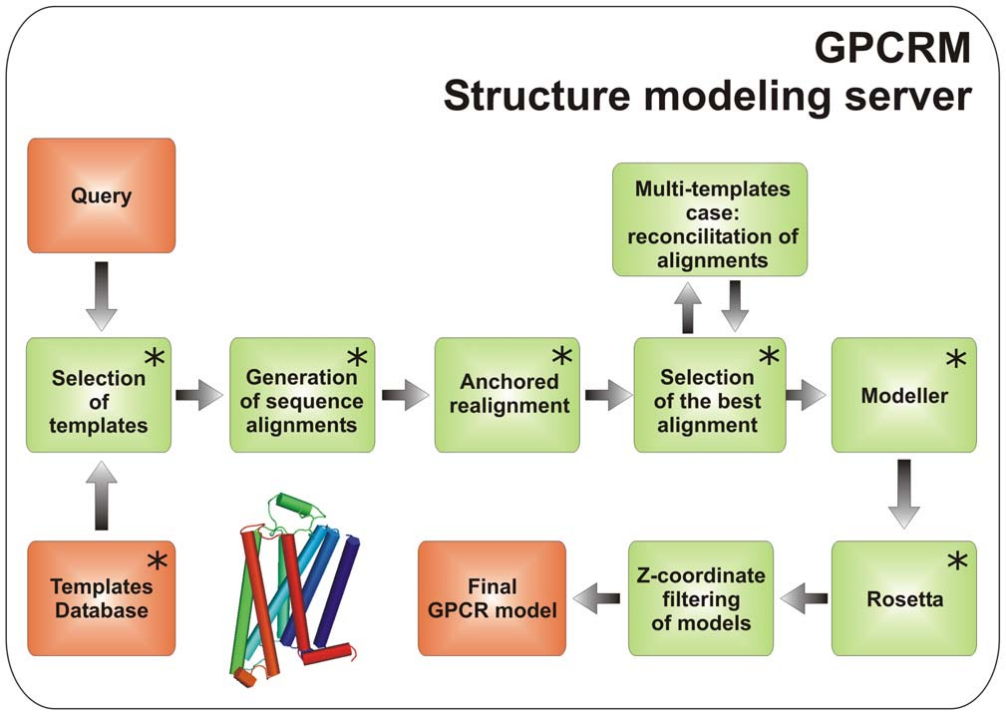
The GPCRM modeling pipeline. A human intervention is possible in the ‘Advanced’ user mode at the steps indicated by asterisks.

The concept of model building from multiple templates was studied extensively by Larsson *et al*. [31] on a large globular protein data set (CASP7 and Wallner's benchmark models) and proved to be successful as long as 2 or 3 templates were used instead of one. On average, further increasing of the number of templates did not improve the protein model and sometimes caused its disruption due to significant structural differences between templates impossible to average by Modeller. GPCRs share a similar 7TM fold which facilitates an efficient averaging of coordinates. For that reason, in the GPCRM pipeline (see Figure 2) a protein model can be built from as many templates as are available using an iterative reconciliation of alignments. What is more, the final protein model is not a sum of structural fragments picked from various templates like in SSFE, but an average structure built on the given set of templates. Such an approach is especially valuable when the selection of the single template is difficult due to low sequence similarity between a modeled GPCR and available templates.

We also implemented in GPCRM two reliable loop modeling methods: Modeller which uses optimization of pseudo-energy function [32], [33] and Rosetta which is based on fragment library [34], [35]. Final GPCRM protein models can be used directly in docking since they contain hydrogen atoms and refined side chains of amino acids. Noteworthy, the activation state of the modeled receptor is taken into account during the model building procedure providing the means for precise docking studies of agonist versus inverse agonist binding to a given GPCR. Generated protein models can be also embedded into the lipid bilayer to perform molecular dynamics (MD) studies of the apo forms of G-coupled receptors optionally containing a lysozyme domain. As the aim of GPCRM is to provide protein models either for docking or MD purposes we did not incorporate any computationally demanding MD simulations. The GPCRM templates database is being constantly updated as new GPCR structures are being released in PDB. The templates data set used in the current study is provided in Table S1 in Supplementary Material S1.

## Results

### Improving the alignment by usage of sequence profiles

As mentioned in Methods, the alignment is generated by GPCRM in three ways: pairwise sequence alignment (PSA), multiple sequence alignment (MSA) and merging of sequence profiles. Here, we compare performance of those methods depending on the ClustalW2 identity score between target and template sequences. Tested protein sequences are from the first data set which includes GPCR structures released before 2012 (for details see ‘Data sets used in the study’ in the Supplementary Material S1). To assess the generated sequence alignments we defined their accuracy as a number of true positives divided by the target sequence length. A ‘true positive’ is the situation in which the same pair of residues (or a residue and a gap) is aligned in the tested alignment as in the reference alignment. The reference sequence alignment shown in Figure S1 in Supplementary Material S1 was computed by VMD from the structural alignment of crystal GPCR structures.

**Figure 3:**
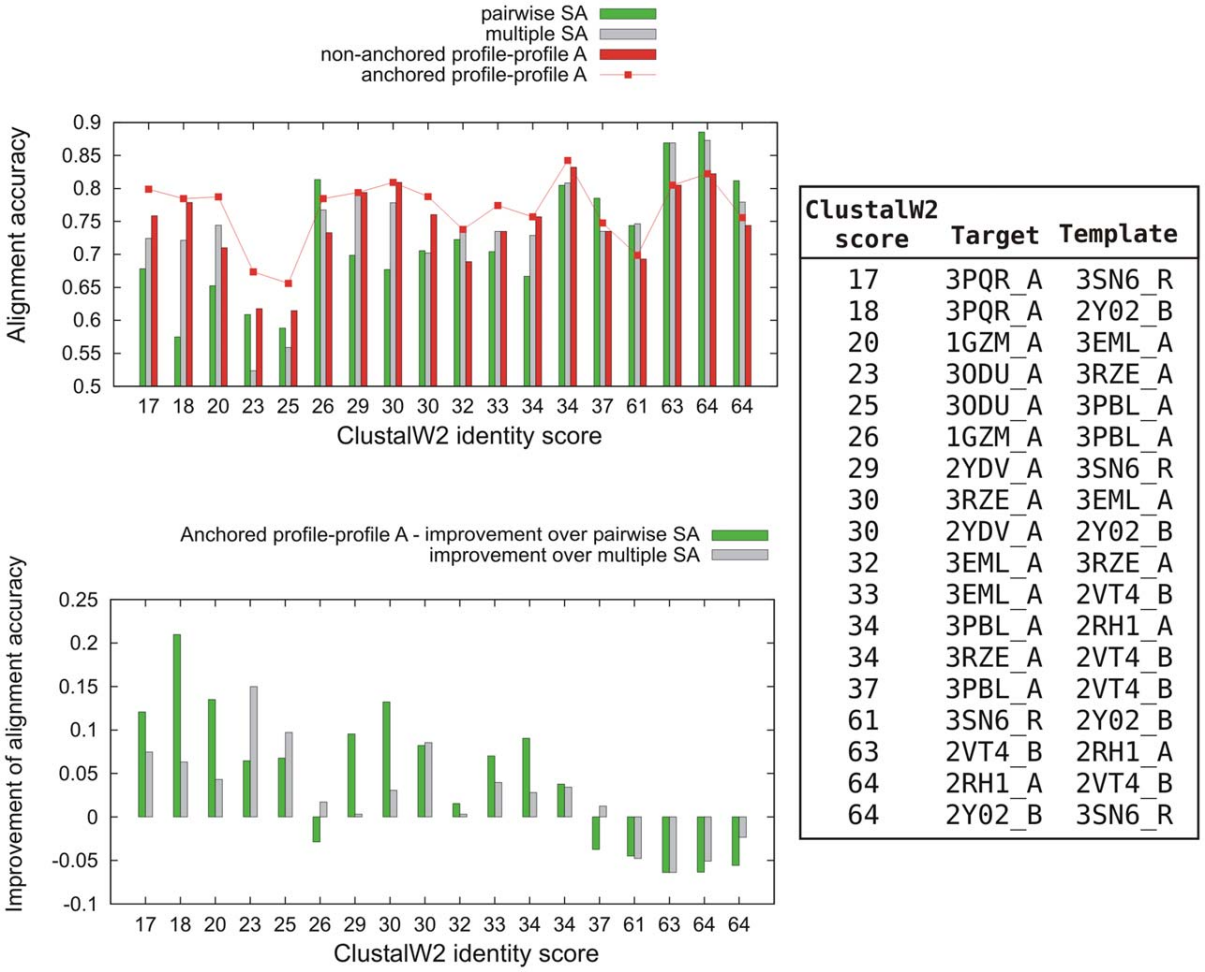
Comparison of various methods for the alignment generation in GPCRM. Here, we plotted ClustalW2 identity scores versus the alignment accuracy (the upper plot) or versus the difference between the accuracy provided by profile-profile alignment and PSA or MSA (the lower plot). The ClustalW2 score and PDB id for both the target and template proteins are provided on the right panel.

As it is shown in Figure 3 (the upper part), the most accurate alignment was produced by a profile-profile comparison. Also the bottom part of Figure 3 clearly shows that the alignment based on either PSA or MSA, as implemented for example in GPCR-Modsim, can be significantly improved by the usage of sequence profiles and the ‘anchored realignment’ step. Nevertheless, a substantial improvement was observed mostly in the area of low sequence identity. Decreased accuracy in the case of high sequence identity can be explained by the fact that additional homologous sequences in the profiles might simply introduce a background noise. Such observations agree with earlier studies on the usage of sequence profiles [23]–[25]. Nevertheless, when the sequence identity was high (over 34% and 60% - see Figure 3), the most accurate alignment (PSA) was easily selected using the GPCRM alignment scoring scheme.

### Improving model quality by using multiple templates

All known GPCR structures have the same 7TMH fold (Figure 1) but the kinks and tilt angles of TM helices may be different which makes the comparative modeling hard. Despite the recent progress [15], a fast and accurate optimization of the orientation angles in TM helices is still a computational challenge which prompted us to develop alternative approaches to comparative modeling of GPCRs such as GPCRM. What is more, the choice of the template structure may significantly influence not only the model quality itself but also the subsequent ligand docking procedure and may lead to false conclusions [36] as was in the case of early homology models of GPCRs based on rhodopsin. The usage of multiple templates in the model building might be a solution to the above problems (see Table 2 - benchmark results). The quality of the final protein model is improved in nearly all cases when another template is used in the modeling. Addition of the third template is more risky as in 6 cases out of 12 tested the final model is slightly inferior to the model produced using one template, confirming earlier studies of [31] on protein classes other than GPCRs. Improvement of the model quality due to the usage of multiple templates is visible in the case of difficult comparative modeling based on low sequence identity. Surprisingly, we have also observed a slight improvement (see Table 2) in the case of high sequence identity when typically only one template is used to build a protein model.

**Table 2:** Comparison of the GPCRM model building procedure based on one, two and three template structures.

Target	Template 1	Template 2	Template 3	RMSD of the binding site area^1^				
				Template 1	Template 2	Template3	Template 1&2	Template 1&2&3
Activated GPCRs structures								
Adenosine A2AR (2YDV_A)	3SN6_R	2Y02_B	3PQR_A	2.82	3.24	5.38	2.82	2.59
	(29)^2^	(30)	(17)					
Rhodopsin (3PQR_A)	2Y02_B	2YDV_A	3SN6_R	4.79	5.11	5.33	5.29	4.94
	(18)	(18)	(17)					
Adrenergic β1AR (2Y02_B)	3SN6	2YDV_A	3PQR_A	1.95	3.92	4.49	2.02	1.84
	(64)	(29)	(17)					
Adrenergic β2AR (3SN6_R)	2Y02	2YDV_A	3PQR_A	2.13	4.62	5.93	2.04	2.39
	(61)	(27)	(15)					
Inactive GPCRs structures								
Rhodopsin (1GZM_A)	3PBL_A	3EML_A	2VT4_B	5.96	6.47	5.47	6.04	5.11
	(26)	(20)	(19)					
Adrenergic β2AR (2RH1_A)	2VT4_B	3PBL_A	3RZE_A	1.41	1.83	2.45	1.28	1.45
	(64)	(35)	(31)					
Adenosine A2AR (3EML_A)	2VT4_B	3RZE_A	2RH1_A	4.20	3.98	4.02	3.30	4.07
	(33)	(32)	(30)					
Chemokine CXCR4 (3ODU_A)	3PBL_A	3RZE_A	2VT4_B	5.51	5.08	6.42	4.79	5.24
	(25)	(23)	(22)					
Dopamine D3R (3PBL_A)	2VT4_B	2RH1_A	3RZE_A	1.73	1.89	2.52	1.81	1.69
	(37)	(34)	(31)					
Histamine H1R (3RZE_A)	2VT4_B	3PBL_A	3EML_A	3.36	3.42	3.62	2.52	2.81
	(34)	(31)	(30)					
Adrenergic β1AR (2VT4_B)	2RH1_A	3PBL_A	3RZE_A	1.45	1.87	3.29	1.14	1.50
	(63)	(38)	(33)					

1 Here, we computed heavy-atoms RMSD of the best model. The binding site area is defined as a set of residues which are in the 5 Å sphere around the ligand in the reference crystal structure.

2 ClustalW2 scores (normalized to 100) indicating sequence identity are provided in brackets.

**Figure 4:**
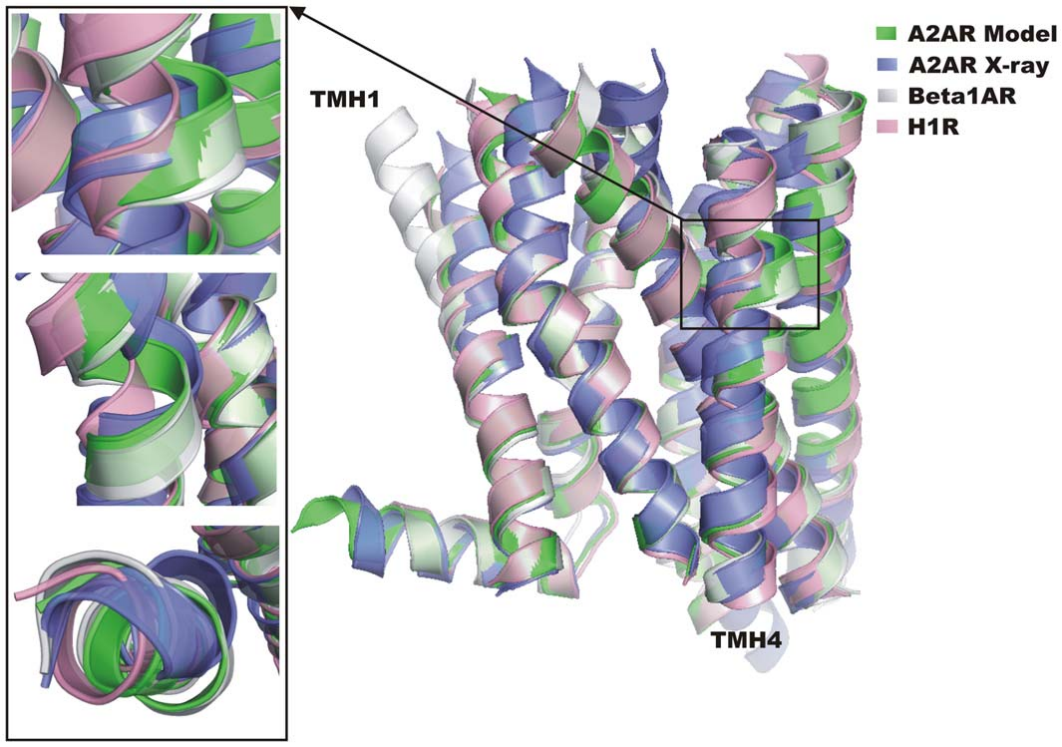
Multiple template modeling of A2AR. The model (green) was generated by GPCRM and is superposed on the crystal structure (blue) and templates used in the model building: the β1AR adrenergic receptor (grey) and the histamine H1R (pink). The bulge observed in TMH4 in β1AR is properly transferred to the A2AR model. Additionally, incorporation of the second template (H1R) improves the kink of TMH1 in the A2A model. The TMH4 bulge can be examined in details in pictures taken from different angles presented on the left.

Detection of bulges and kinks in TM helices is crucial for the GPCR structure modeling. In the data set used in the study there are two examples in which we could test modeling of bulges using GPCRM. The first example is modeling of the adenosine A2A receptor (A2AR) structure (PDB id: 3EML) based on β1AR (PDB id: 2VT4) and the histamine H1 receptor (H1R) (PDB id: 3RZE). There is a small bulge in TMH4 in β1AR which is not present in the case of histamine H1R. GPCRM correctly predicts a necessary gap in the alignment (Figure S2 in Supplementary Material S1) and produces a proper deformation of TMH4 in the form of a bulge (Figure 4). Although the shape of this bulge is not exactly the same as in the crystal structure of the A2AR, because it fits the coordinates of one of the templates (β1AR), its presence preserves the rest of TMH4 from taking the wrong orientation. Nevertheless, if we used only one, the most similar template with the helical bulge inside the TMH4 (β1AR), another helix (TMH1) would be kinked in the opposite direction to that in the crystal structure of A2AR. Due to the usage of the second, less similar template (H1R) the kink direction in TMH1 had been improved (Figure 4). The second example of the proper bulge detection in the GPCRM automatic mode is modeling of the κ-opioid receptor based on the CXCR4 chemokine receptor and histamine H1R (Figures S3, S4 and S5 in Supplementary Material S1). This time a bulge was not introduced in TMH2 (although present in the H1R template) in agreement with the crystal structure of the κ-opioid receptor. Based on the above two examples of A2AR and the κ-opioid receptor we conclude that GPCRM is able to either properly introduce or remove a structural bulge in transmembrane helices due to the usage of multiple templates instead of one template structure.

Incorporation of the additional template structures is also valuable while building a protein model based on the CXCR4 chemokine receptor (3ODU) since the common helix H8 is not present in this structure due to slightly different amino acid composition of that C-terminal region when compared with other GPCR structures known to date [37]. A straightforward method to include helix H8 in the model would be to use another GPCR template containing helix H8. Such simple solution should be possible due to the exceptional features of the Modeller algorithm in which spatial restraints are converted to probability density functions with the regular secondary structure preferred. Therefore, the Modeller program is able to build a model based on even ambiguous or inconsistent spatial restraints derived from various protein templates. To test that hypothesis, we used GPCRM (Modeller only) to generate the human dopamine D3R model (PDB id: 3PBL) in two ways: firstly, using 2VT4_B and 3OE6_A templates separately and secondly, together. The C-alpha RMSD of helix H8 with respect to the native structure was 0.53Å (template: 2VT4_B - β1AR), 7.60Å (template: 3OE6_A - CXCR4) and 0.59Å (templates: 2VT4_B and 3OE6_A). Adding another template with H8 (2VT4_B) to CXCR4 during the model building resulted in decrease of RMSD and thus confirmed our hypothesis. Such solution can safely be used in the model building based on the CXCR4 receptor.

### Overall GPCRM performance in model building and docking

**Figure 5:**
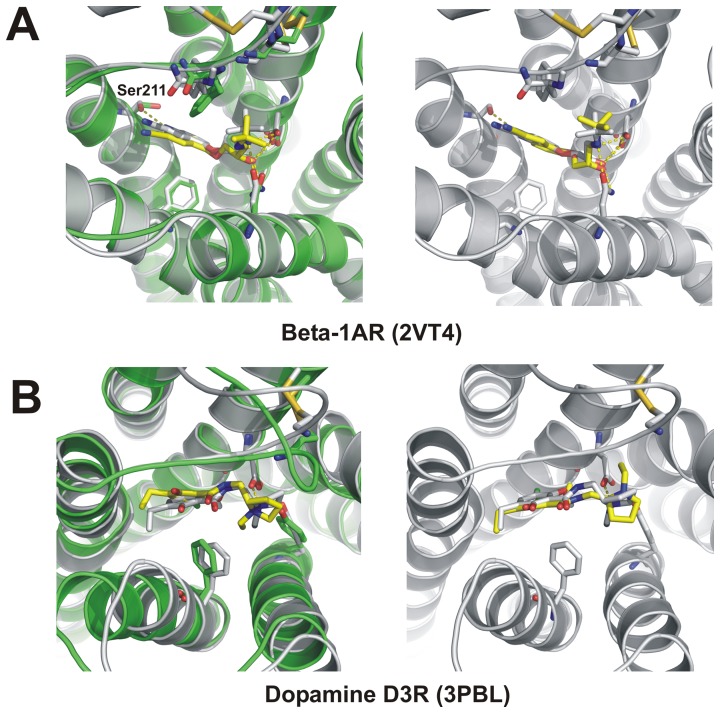
Antagonist docking to GPCRM-generated homology models versus self-docking: β1AR receptor (A) and D3R receptor (B). Structures of complexes with indicated polar contacts obtained by crystallography are shown in grey, while the docked structures are depicted in yellow. GPCRM-generated homology models are shown in green. Left panels show the best poses obtained in the docking to corresponding protein homology models. Right panels show results of self-docking to crystallographic structures (PDB id: 2VT4 and 3PBL). All polar contacts were preserved, except one hydrogen bond with Ser211 (A).

In a typical high-throughput virtual screening several thousand of various compounds are docked to a receptor structure. Such a large number imposes limitations on the docking precision and conformational sampling. Therefore, for testing the usefulness of GPCRM in drug design studies we have chosen fast, standard precision, flexible-ligand and rigid receptor docking in Glide with the default force field settings. The obtained results were compared to a self-docking test on the crystal structures of GPCRs performed by Glide with the same force field settings. The quality of the Rosetta-generated models seems to be sufficient to use them in virtual screening as the best (of the lowest RMSD) ligand poses (Table 3) contained properly oriented ligands in the binding site (Figure 5). In general the prediction of GPCR ligand binding modes is very challenging since even in the easy case of the self-docking to crystal structures not all the ligand rings are positioned properly (Figure S6–right panels in Supplementary Material S1). Most of rotamers of amino acids were properly predicted by GPCRM preserving polar contacts most important for the ligand binding. Nevertheless, falsely predicted rotamers of Thr112 (Figure S6–B in Supplementary Material S1) and Asp97 (Figure S6–C in Supplementary Material S1) caused a slight movement of ligands, yet preserving their proper orientation. In general, the quality of binding sites as well as the overall GPCR structures were much better (lower RMSD) in the case of loop modeling by Rosetta than by Modeller (Table 3). On average, the binding site area had been improved after the Rosetta step by 1–2 Å. Interestingly, the final Rosetta refinement slightly improved the rotamers in the TM region even though the protein backbone was restrained.

**Table 3:** Benchmark results of GPCRM in structure modeling and small molecule docking.

PDB id	Modeller			Rosetta						Reference:self-docking^1^	
	C-alpha	Heavy-atoms RMSD		C-alpha	Heavy-atoms RMSD						
	RMSD			RMSD							
	Full model	TM region	Binding site^2^	Full model	TM region	Binding site		The best ligand pose			
Activated GPCRs structures											
Adenosine A2AR (2YDV_A)	5.68	4.34	3.89	5.89	3.82	3.73			3.27	0.42	
Rhodopsin (3PQR_A)	10.13	5.29	5.01	6.21	4.06	4.79			2.19	2.70	
Adrenergic β1AR (2Y02_B)	3.91	4.74	3.14	4.40	4.33	1.86			1.47	0.80	
Adrenergic β2AR (3SN6_R)	3.49	4.25	3.95	3.42	3.33	2.49			1.79	1.06	
Inactive GPCRs structures											
Rhodopsin (1GZM_A)	12.21	5.43	6.45	5.46	2.84	5.87			3.13	0.94	
Adrenergic β2AR (2RH1_A)	2.39	3.13	2.99	2.44	1.59	1.71			1.18	0.64	
Adenosine A2AR (3EML_A)	3.23	3.88	4.16	3.20	2.79	3.77			22.78	2.71	
Chemokine CXCR4 (3ODU_A)	4.31	4.47	5.49	4.08	3.63	4.86			4.09	1.02	
Dopamine D3R (3PBL_A)	2.55	3.40	3.16	2.24	2.17	2.22			1.25	1.06	
Histamine H1 (3RZE_A)	2.61	3.41	3.94	2.55	2.13	2.88			2.64	0.70	
Adrenergic β1AR (2VT4_B)	2.36	3.15	3.01	2.15	1.46	1.60			0.73	0.63	

1 Here, we provided as a reference results of self-docking to crystal structures of GPCRs.

2 The binding site area is defined as a set of residues which are inside the 5Å sphere around the ligand.

Although some attempts have been made in the field of GPCR modeling from multiple templates [38], [39], proving importance of such approach [40], GPCRM is the first method which implements the concept efficiently without any limitations concerning the number of templates used and their sequence as well as structural similarities to each other. A possibility of manual adjustment of the multiple template alignment online, that is offered by GPCRM, is sometimes inevitable as it was evident in the case of opioid receptor modeled based on squid rhodopsin, turkey β1-, human β2-adrenoreceptors and bovine rhodopsin [41]. In some cases, given a quite accurate alignment in which nearly 90% of residues are correctly aligned (β1AR (2VT4) and β2AR (2RH1) - see Figure 3), it may be beneficial to skip the loop refinement step and rely only on a template structure. Indeed, C-alpha RMSD of the best basic Modeller model (without loop modeling) for the β1AR case was 1.27Å and for the best loop model: 1.77Å. GPCRM provides an option to skip the loop refinement in the advanced user mode.

### Comparison with other methods

In the year 2012 several new structures of GPCRs were released. At that time, GPCRM has already been at the internal tests stage so we decided to compare our preliminary results with the current performance of other methods. However, the provided protein models frequently contained a lysozyme domain, mutated residues or were deprived of loops. Therefore, we used a TM-align program [42] to compute the TM-score and C-alpha RMSD with respect to the crystal structures of GPCRs. TM-score is frequently used to assess the performance of various protein structure prediction methods when RMSD fails to detect the best protein model [43]. At the time of the benchmarking the GPCRM templates database consisted only of the GPCRs structures released till the end of 2011. Results presented in Table S2 in Supplementary Material S1 clearly show that GPCRM performs quite well in comparison with the currently available methods, including the recently published GPCR-Modsim. Unfortunately, the fast update of templates database prevented us from including the well-known GPCR-ITASSER in the comparison.

In three cases out of four, GPCRM provided the best GPCR model (Table S2 in Supplementary Material S1). Only in the case of muscarinic M2R (3UON) the database of GPCR protein models generated by Yasara (GPCRDB) provided a better protein model than GPCRM. The main factor which explains differences between the results provided in Table S2 in Supplementary Material S1 is the templates data set which was used by each method. For example, the highest difference between GPCRM and other methods (improvement of the TM-score of about 0.260 with respect to the best model which was provided by GPCRDB) was observed in the case of 4DAJ (muscarinic M3R). Even after removing the loops, the GPCRM-generated model was still better than the one generated by GPCRDB (TM-score: 0.769 and 0.736, respectively) which proves that the difference between models concerned not only the loops but also the TM region defined strictly by the coordinates of the templates. As it was mentioned in [13] a selection of templates is crucial due to the existence of various deformations of TM helices (bulges and kinks) which are not always present in the same place in every GPCR structure. In the case of the κ-opioid receptor (4DJH) GPCRM properly predicted the lack of a bulge in TMH2 (Figures S3 and S4 in Supplementary Material S1) while the other methods did not (Figure S5 in Supplementary Material S1). The reason was again the templates used by GPCRM (histamine H1R and chemokine CXCR4) and other methods (rhodopsin and β1AR).

If we used the templates which were used by other methods results would be obviously slightly worse yet not in all cases, e.g., M2R (3UON) based on 2VT4, 3V2Y based on 3EML and 4DJH based on 2VT4, 2RH1, 3EML and 1U19 (see Table S3 in Supplementary Material S1). What is more, the averaging of templates structures in the model building, as implemented in GPCRM, seems to be a better approach than the one implemented in SSFE (compare Table S2 in Supplementary Material S1 and the last column of Table S3 in Supplementary Material S1). As we mentioned before, GPCRM uses coordinates of many templates simultaneously and the final model is the weighted average of the input structures. Such functionality is similar to that of GPCR-I-TASSER but different from SSFE which produces a GPCR model as a sum of TM helices taken from different templates. Nevertheless, in the GPCRM advanced mode the alignment might be manually changed by a user to produce GPCR models in a similar fashion as SSFE.

In Table S2 in Supplementary Material S1 we divided GPCRM results into: GPCRM-Modeller and GPCRM-Rosetta to show differences between these two loop modeling methods (Modeller and Rosetta). The TM core is the same in both cases. Interestingly, in the case of 3V2Y (the lipid receptor) only the GPCRM-Rosetta models were better than models generated by other methods. It showed that the good performance of GPCRM is not only due to the selection of templates but also due to the extended loop protocol which incorporates Rosetta and the Z-coordinate based filtering of models. It is worth to mention that, even if the loops in GPCR are too long for a reliable prediction, GPCRM always provides a complete protein model with the full sequence the user has submitted. That facilitates the usage of the GPCRM-generated models straightforwardly in, for example, Monte Carlo simulations in which very long loops or domains can be folded into the native-like structures (performed by e.g. CABS [44], UNRES [45] or I-TASSER [46]).

## Conclusions

We have provided the scientific community with a new approach to structure modeling of GPCRs with an easy access online. Our method satisfies the requirements indispensable for *in silico* drug discovery and provides reliable GPCR models as was proved by benchmarking currently available methods. Although the usage of multiple templates in GPCR structure modeling was probed earlier on small data sets [39]–[41], the current study is the first that shows results for several GPCRs and confirms the reliability and usefulness of such modeling in drug discovery. What is more, previous studies of the GPCR activation mechanism involved homology models based on only one template (e.g. rhodopsin) which often led to biased conclusions [47]. Usage of multiple templates in the GPCRM modeling pipeline could lead to more certain conclusions regarding docking and research on GPCR activation. GPCRM is not only a new protein structure prediction method but also an integrated online platform. It was designed to significantly decrease the time of structure generation and analysis needed in large scale biological projects. The platform can be used not only by computational biologists but also experimentalists to visualize their findings on theoretical models. In our database we have deposited the precomputed GPCR models of the members of the Rhodopsin-like class, which were built using the currently available templates of GPCRs and a multilevel approach presented in this manuscript. Although GPCRM has been developed for the Rhodopsin-like class its usefulness is not limited only to that class because the implemented sequence profiles can facilitate studies of distantly related proteins.

## Methods

### The GPCRM pipeline description

GPCRM is the first method for modeling GPCR structures which integrates various approaches for template detection, alignment generation, model building, loop refinement and model filtering based on the Z-coordinate, with the optional human intervention almost at every stage (see Figure 2 and Supplemental Methods in Supplementary Material S1). To adequately model distantly related GPCRs in a so-called ‘twilight zone’ of low sequence similarity [48] we fitted the number of selected templates used in the modeling to the level of sequence similarity to the target. Namely, when a sequence similarity is low then a GPCR model can be built on a set of template structures (2, by default) which are translated into spatial restraints and efficiently averaged with a subsequent step of all-atom minimization to provide protein-like coordinates. The alignment generation step includes a profile comparison procedure which is much more efficient than a simple alignment of two protein sequences. Additionally, GPCRM incorporates a Z-coordinate based filter to generate only such GPCR models in which extra and intracellular loops as well as N and C-termini do not enter the membrane. Such a filter had to be applied because neither Rosetta nor Modeller original loop protocols include any information about the location of a protein with respect to the membrane. Incorporating two procedures for loop modeling: fragment-based (Rosetta) and energy minimization-based (Modeller), GPCRM can overcome limitations of each of those approaches alone which are: completeness of the fragment database and convergence of optimization procedures[33]. What is more, GPCRM slightly improves the Rosetta loop modeling through the use of GPCR-specific cut-points (see Supplemental Methods in Supplementary Material S1).

The modeling procedure begins with aligning a target sequence against all template sequences in the GPCRM database of templates (see the Supplementary Material S1) using MUSCLE [49] and ClustalW2 [50]. If the ClustalW2 score is above 50, a single template is selected to build a protein model, otherwise two, most similar templates are chosen. In the next step, close homologous sequences are found by BLAST and used for a precise target-template alignment generation. During the alignment generation step we used a BLOSUM62 substitution matrix for alignment scoring, though there are other substitution matrices derived specifically for membrane proteins e.g. PHAT [51], JTT [52], or SLIM [53], which are excellent for detection of distant sequence homologs. Nevertheless, it has not yet been proved that any of those membrane proteins-specific matrices is significantly better in simultaneously scoring both the globular loops and TM regions in the alignments, which is the case in our study, without a complete switch to a bipartite alignment method [54]. The sequence alignment, template structures and optional information about the conserved disulfide bond between EC2 (the second extracellular loop) and TMH3 are passed to Modeller. The best 10 models according to a DOPE scoring function are then selected for a loop refinement in Rosetta. Finally, hydrogen atoms are added to the models and a short refinement in the all-atom Rosetta force field is performed. The best 10 models according to Rosetta all-atom total energy are provided as the final result. GPCRM offers also the possibility to incorporate a lysozyme molecule inside the model. Such lysozyme-fused GPCR models may be useful, for example, in molecular replacement for processing low resolution X-ray data [55] or in MD simulations [56], [57]. GPCRM integrates a number of our programs (depicted in Figure 2 as ‘Selection of templates’, ‘Generation of sequence alignments’, ‘Anchored realignment’, ‘Reconciliation of multiple template alignments’, ‘Selection of the best alignment’ and ‘Z-coordinate based filtering of models’) with six well-known academic programs: MUSCLE, CLUSTALW2, BLAST, Modeller, Rosetta and PyMOL. GPCRM was implemented in Python using Biopython libraries [58] with the user interface based on the Django web framework with the Jmol java applet [59]. A detailed description of alignment generation, model building and loop modeling procedures in GPCRM is provided in Supplementary Material S1.

## Supporting Information

Supplementary Material S1 This file contains: Data sets used in the study, Tables S1-S3, Figures S1-S5 and Supplemental Methods. **Table S1.** The templates data set used in the current study. **Table S2.** GPCRs released in 2012 - benchmark results of web services in GPCR structure modeling. **Table S3.** GPCRs released in 2012 - benchmark results of GPCRM in GPCR structure modeling depending on the templates data set. **Figure S1.** **The reference sequence alignment of GPCRs.** The alignment was generated by VMD (a MultiSeq plugin [22]) based on the structural alignment of GPCRs of known 3D structures. Positions of highly conserved residues are marked according to Ballesteros-Weinstein numbering scheme. Positions of TM helices based on rhodopsin (1GZM) are marked with grey. **Figure S2.** **The sequence alignment used in GPCRM modeling of A2AR.** A fragment which corresponds to the bulge in TMH4 is marked by a square box. The template with the bulge in the structure (2VT4 – β1AR) is aligned against the target sequence (A2AR) without any gaps in that fragment while the template without the bulge (3RZE – H1R) is aligned with a one-residue gap. **Figure S3.** **The sequence alignment used in GPCRM modeling of κ-opioid receptor.** A fragment which corresponds to the lack of bulge in TMH2 is marked in the alignment (a square box). The template without the TMH2 bulge (3ODU – CXCR4) is aligned against the target sequence (κ-opioid receptor) without any gap in that fragment, while the template with the TMH2 (3RZE – H1R) bulge is aligned with a one-residue gap. **Figure S4. The model of κ-opioid receptor (PDB id: 4DJH).** The model (green) was generated by GPCRM and superposed on the crystal structure (blue) and templates used in the model building: the histamine H1R (grey) and the CXCR4 receptor (pink). The bulge observed in TMH2 in H1R was removed and was not transferred to the κ-opioid model. Nevertheless, averaging of H1R and CXCR4 coordinates in TMH1 did not result in the proper kink of TMH1 proving limitations of the Modeller software. **Figure S5.** **Models of κ-opioid receptor (4DJH) generated by currently available methods.** All models are superposed on the crystal structure (blue). The bulge in TMH2 which is not present in the crystal structure is depicted. Templates used in the model building by each method are as follows: rhodopsin (ModWeb/ModBase), β1AR (GPCRDB and GPCR-Modsim), β1AR together with β2AR, A2A and rhodopsin (SSFE). **Figure S6.** **Ligand docking to GPCRM-generated homology models versus self-docking: β2AR (A), H1R (B), CXCR4 (C) and metarhodopsin II (D).** The reference crystal complexes with indicated polar contacts (yellow dashed lines) are shown in grey, while the docked ligand poses are depicted in yellow. GPCRM-generated homology models of receptors are shown in green. Left panels show the best poses obtained from docking to corresponding protein homology models. Right panels show results of self-docking to crystal structures (PDB id: 3SN6, 3RZE, 3ODU, 3PQR). Most polar contacts were preserved except for: Ser203 (A), Thr112 (B), Asp97 (C). Although Ile189 and Tyr191 in the EC2 loop are not as deep in the binding pocket as in the crystal structure of metarhodopsin II (D), retinal was positioned in the homology model with the proper orientation of the β-ionone ring (left panel) contrary to the self-docking results (right panel).
(DOCX)
