# Supplementary material for: Towards Improved Quality of GPCR Models by Usage of Multiple Templates and Profile-Profile Comparison
Source: PLoS One. 2013 Feb 28;8(2):e56742. doi: 10.1371/journal.pone.0056742 (PMC3585245; doi:10.1371/journal.pone.0056742)
Supplement: Supplementary Material S1 — This file contains: Data sets used in the study, Tables S1-S3, Figures S1-S5 and Supplemental Methods. Table S1. The templates data set used in the current study. Table S2. GPCRs released in 2012 - benchmark results of web services in GPCR structure modeling. Table S3. GPCRs released in 2012 - benchmark results of GPCRM in GPCR structure modeling depending on the templates data set. Figure S1. The reference sequence alignment of GPCRs. The alignment was generated by VMD (a MultiSeq plugin [22]) based on the structural alignment of GPCRs of known 3D structures. Positions of highly conserved residues are marked according to Ballesteros-Weinstein numbering scheme. Positions of TM helices based on rhodopsin (1GZM) are marked with grey. Figure S2. The sequence alignment used in GPCRM modeling of A2AR. A fragment which corresponds to the bulge in TMH4 is marked by a square box. The template with the bulge in the structure (2VT4 – β1AR) is aligned against the target sequence (A2AR) without any gaps in that fragment while the template without the bulge (3RZE – H1R) is aligned with a one-residue gap. Figure S3. The sequence alignment used in GPCRM modeling of κ-opioid receptor. A fragment which corresponds to the lack of bulge in TMH2 is marked in the alignment (a square box). The template without the TMH2 bulge (3ODU – CXCR4) is aligned against the target sequence (κ-opioid receptor) without any gap in that fragment, while the template with the TMH2 (3RZE – H1R) bulge is aligned with a one-residue gap. Figure S4. The model of κ-opioid receptor (PDB id: 4DJH). The model (green) was generated by GPCRM and superposed on the crystal structure (blue) and templates used in the model building: the histamine H1R (grey) and the CXCR4 receptor (pink). The bulge observed in TMH2 in H1R was removed and was not transferred to the κ-opioid model. Nevertheless, averaging of H1R and CXCR4 coordinates in TMH1 did not result in the proper kink of TMH1 proving limitations of the Modeller soft [file pone.0056742.s001.docx]

**­­­­Towards improved quality of GPCR models by usage of multiple templates and profile-profile comparison**

Dorota Latek^1^*, Pawel Pasznik^1^, Teresa Carlomagno^2^, Slawomir Filipek^3^*

**Supplementary material**

Table of content

1. Data sets used in the study
2. Tables S1-S3
3. Figures S1-S5
4. Supplemental Methods

**Data sets used in the study**

For the purpose of the current study we used 20 high or medium resolution templates (Table S1) of unique GPCRs which are divided into two groups: inactive (1GZM_A, 1U19_A, 2RH1_A, 2VT4_B, 3EML_A, 3ODU_A, 3PBL_A, 3RZE_A, 4DAJ_A, 4DJH_A, 4DKL_A, 4EJ4_A, 3UON_A, 3V2Y_A, 4EA3_A) and actived or semi-actived receptors (2Y02_B, 2YDV_A, 3PQR_A, 3QAK_A, 3SN6_R). That templates subset which was available in the GPCRM database during the manuscript preparation is being constantly updated (see <http://gpcrm.biomodellab.eu/news>). That first data set, which was composed of high or reasonable resolution structures selected from all unique GPCRs released in PDB before the year 2012, was further splitted into seven inactive receptor structures (bound to agonists) and four active or semi-activated receptor structures (bound to inverse agonist or antagonists). As it was discussed in [[1](#_ENREF_1)] not all PDB structures of receptors bound to agonists capture their fully activated state due to experimental difficulties to stabilize such a dynamic state. Therefore, we used the above term ‘semi-activated’.

The second test set included four inactive receptors structures released in 2012 (no structures with agonists were obtained) and was used to compare the performance of GPCRM with other servers for the structure prediction of members of the Rhodopsin-like class. We also prepared a third set of 102 human GPCRs form the Rhodopsin-like class which were selected for crystallization in the next few years in the Stevens group [[2](#_ENREF_2)]. That test was run in full automatic mode, except few the most difficult cases, yet with the fast loop modeling. It was used to test the GPCRM performance and to provide to a research community a database of precomputed full-length GPCR models. They are available to download from <http://gpcrm.biomodellab.eu>.

**Tables**

**Table S1. The templates data set used in the current study.**

| PDB id | Name | Reference |
| --- | --- | --- |
| 1GZM_A | bovine rhodopsin | [[3](#_ENREF_3)] |
| 1U19_A | bovine rhodopsin | [[4](#_ENREF_4)] |
| 2RH1_A | human β_2_ adrenergic receptor | [[5](#_ENREF_5)] |
| 2VT4_B | human β_1_ adrenergic receptor | [[6](#_ENREF_6)] |
| 2Y02_B | turkey β_1_ adrenergic receptor | [[7](#_ENREF_7)] |
| 2YDV_A | human adenosine A_2_A receptor | [[1](#_ENREF_1)] |
| 3EML_A | human adenosine A_2_A receptor | [[8](#_ENREF_8)] |
| 3ODU_A | human CXCR4 chemokine receptor | [[9](#_ENREF_9)] |
| 3PBL_A | human dopamine D_3_ receptor | [[10](#_ENREF_10)] |
| 3PQR_A | bovine rhodopsin - metarhodopsin II | [[11](#_ENREF_11)] |
| 3QAK_A | human adenosine A_2_A receptor | [[12](#_ENREF_12)] |
| 3RZE_A | human histamine H_1_ receptor | [[13](#_ENREF_13)] |
| 3SN6_R | human β_2_ adrenergic receptor | [[14](#_ENREF_14)] |
| 4DAJ_A | rat M_3_ muscarinic acetylcholine receptor | [[15](#_ENREF_15)] |
| 4DJH_A | human κ opioid receptor | [[16](#_ENREF_16)] |
| 4DKL_A | mouse μ opioid receptor | [[17](#_ENREF_17)] |
| 4EJ4_A | mouse δ opioid receptor | [[18](#_ENREF_18)] |
| 3UON_A | human M_2_ muscarinic acetylcholine receptor | [[19](#_ENREF_19)] |
| 3V2Y_A | sphingosine 1-phosphate receptor (lipid receptor) | [[20](#_ENREF_20)] |
| 4EA3_A | human nociception / orphanin FQ peptide receptor | [[21](#_ENREF_21)] |

**Table S2. GPCRs released in 2012 - benchmark results of web services in GPCR structure modeling.**

| Assessment functions  & templates used | GPCRM^1^ | GPCRM | ModWeb/  ModBase | GPCRDB | GPCR-ModSim | SSFE |
| --- | --- | --- | --- | --- | --- | --- |
|  | Rosetta | Modeller |  |  |  |  |
| Human muscarinic M2R (3UON) | | | | | | |
| TM-score | 0.884 | 0.876 | 0.891 | 0.900 | 0.830 | 0.724 |
| RMSD^2^ | 2.43 | 2.39 | 2.48 | 2.04 | 2.91 | 1.90 |
|  | (274) | (270) | (274) | (270) | (265) | (217) |
| Templates | 3RZE_A^3^ (37)^4^ | 3RZE_A (37) | 2RH1_A (26) | 2VT4_B (30) | 3PBL_A (31) | 2VT4_B (30) |
|  | 3PBL_A (31) | 3PBL_A (31) |  |  |  | 2RH1_A (26) |
|  |  | |  |  |  | 3EML_A (25) |
|  |  | |  |  |  | 1U19_A (20) |
| Lipid receptor (3V2Y) | | | | | | |
| TM-score | 0.802 | 0.772 | 0.801 | 0.801 | 0.800 | 0.661 |
| RMSD | 2.81 | 2.74 | 3.03 | 2.78 | 2.81 | 2.12 |
|  | (272) | (260) | (277) | (263) | (262) | (213) |
| Templates | 3RZE_A (25) | 3RZE_A (25) | 3C9L_A (16) | 3EML_A (25) | 3EML (25) | 2VT4_B (26) |
|  | 3EML (25) | 3EML (25) |  |  |  | 2RH1_A (21) |
|  |  | |  |  |  | 2Z73_A (14) |
|  |  | |  |  |  | 1U19_A (16) |
| Rat muscarinic M3R (4DAJ) | | | | | | |
| TM-score | 0.902 | 0.897 | X-ray structure already included | 0.656 | 0.856 | 0.707 |
| RMSD | 2.30 | 2.47 |  | 3.06 | 2.97 | 2.39 |
|  | (265) | (264) |  | (255) | (262) | (212) |
| Templates | 3RZE_A (37) | 3RZE_A (37) |  | 2VT4_B (33) | 2RH1_A (33) | 2RH1_A (33) |
|  | 2RH1_A (33) | 2RH1_A (33) |  |  |  | 3EML_A (26) |
|  |  | |  |  |  | 2Z73_A (21) |
| Human κ-opioid receptor (4DJH) | | | | | | |
| TM-score | 0.857 | 0.857 | 0.812 | 0.793 | 0.736 | 0.694 |
| RMSD | 3.02 | 3.29 | 3.43 | 3.23 | 3.28 | 1.96 |
|  | (283) | (285) | (275) | (266) | (250) | (215) |
| Templates | 3RZE_A (29) | 3RZE_A (29) | 1GZM_A (17) | 2VT4_B (23) | 2VT4_B (23) | 2VT4_B (23) |
|  | 3ODU_A (25) | 3ODU_A (25) |  |  |  | 2RH1_A (24) |
|  |  | |  |  |  | 3EML_A (21) |
|  |  | |  |  |  | 1U19_A (17) |
|  |  | |  |  |  | 2Z73_A (21) |

^1^The templates database of GPCRM consisted of GPCRs structures released till the end of 2010.

^2^ A number of residues which were used to compute RMSD is provided in brackets.

^3^ Here, we provided PDB id for the following GPCRs: 3RZE – histamine H1R, 3PBL – dopamine D3R, 2RH1 – adrenergic β2AR, 1U19 – bovine rhodopsin, 3EML – adenosine A2AR, 3C9L – bovine rhodopsin, 2Z73 - squid rhodopsin, 3ODU – chemokine CXCR4, 1GZM – bovine rhodopsin, 2VT4 – adrenergic β1AR.

^4^ ClustalW2 scores (normalized to 100) indicating sequence identity are provided in brackets.

**Table S3. GPCRs released in 2012 - benchmark results of GPCRM in GPCR structure modeling depending on the templates data set.**

| Assessment functions  & templates used | GPCRM – a full protein model | | | | GPCRM – a protein model without loops | |
| --- | --- | --- | --- | --- | --- | --- |
| Human muscarinic M2R (3UON) | | | | | |  |
| TM-score | 0.816 | 0.900 | 0.804 | 0.824 | 0.937 | |
| RMSD^1^ | 2.34 | 2.50 | 2.51 | 2.24 | 1.53 | |
|  | (275) | (278) | (274) | (277) | (217) | |
| Templates^2^ | 2RH1_A (26) | 2VT4_B (30) | 3PBL_A (31) | 2VT4_B (30) | 2VT4_B (30) | |
|  |  |  |  | 2RH1_A (26) | 2RH1_A (26) | |
|  |  |  |  | 3EML_A (25) | 3EML_A (25) | |
|  |  |  |  | 1U19_A (20) | 1U19_A (20) | |
| Corresponding method^3^ | ModWeb/ModBase | GPCRDB | GPCR-Modsim | SSFE | SSFE | |
| Lipid receptor (3V2Y) | | | | | |  |
| TM-score |  | 0.768 | 0.805 | 0.807 | 0.903 | |
| RMSD |  | 3.63 | 2.80 | 2.80 | 1.90 | |
|  |  | (279) | (272) | (277) | (216) | |
| Templates |  | 1U19_A (16) | 3EML_A() | 2VT4_B (26) | 2VT4_B (26) | |
|  |  |  |  | 2RH1_A (21) | 2RH1_A (21) | |
|  |  |  |  | 1U19_A (16) | 1U19_A (16) | |
| Corresponding method |  | ModWeb/ModBase | GPCRDB | SSFE | SSFE | |
|  |  |  | GPCR-Modsim |  |  | |
| Rat muscarinic M3R (4DAJ) | | | | | |  |
| TM-score |  | 0.894 | 0.895 | 0.900 | 0.906 | |
| RMSD |  | 2.48 | 2.41 | 2.26 | 1.91 | |
|  |  | (266) | (266) | (266) | (213) | |
| Templates |  | 2RH1_A (33) | 2VT4_B | 2RH1_A (33) | 2RH1_A (33) | |
|  |  |  |  | 3EML_A (26) | 3EML_A (26) | |
|  |  |  |  | 1GZM_A (21) | 1GZM_A (21) | |
| Corresponding method |  | GPCR-Modsim | GPCRDB | SSFE | SSFE | |
| Human κ-opioid receptor (4DJH) | | | | | |  |
| TM-score |  | 0.833 | 0.840 | 0.868 | 0.906 | |
| RMSD |  | 3.27 | 3.17 | 2.96 | 2.13 | |
|  |  | (283) | (280) | (286) | (215) | |
| Templates |  | 1GZM_A (17) | 2VT4_B (23) | 2VT4_B (23) | 2VT4_B (23) | |
|  |  |  |  | 2RH1_A (24) | 2RH1_A (24) | |
|  |  |  |  | 3EML_A (21) | 3EML_A (21) | |
|  |  |  |  | 1U19_A (17) | 1U19_A (17) | |
| Corresponding method |  | ModWeb/ModBase | GPCRDB | SSFE | SSFE | |
|  |  |  | GPCR-Modsim |  |  | |

^1^ A number of residues which were used to compute RMSD is provided in brackets.

^2^ ClustalW2 scores (normalized to 100) indicating sequence identity are provided in brackets.

^3^ Results of the corresponding methods are presented in Table S2.

**Figures**

**Figure S1.** **The reference sequence alignment of GPCRs.** The alignment was generated by VMD (a MultiSeq plugin [[22](#_ENREF_22)]) based on the structural alignment of GPCRs of known 3D structures. Positions of highly conserved residues are marked according to Ballesteros-Weinstein numbering scheme. Positions of TM helices based on rhodopsin (1GZM) are marked with grey.


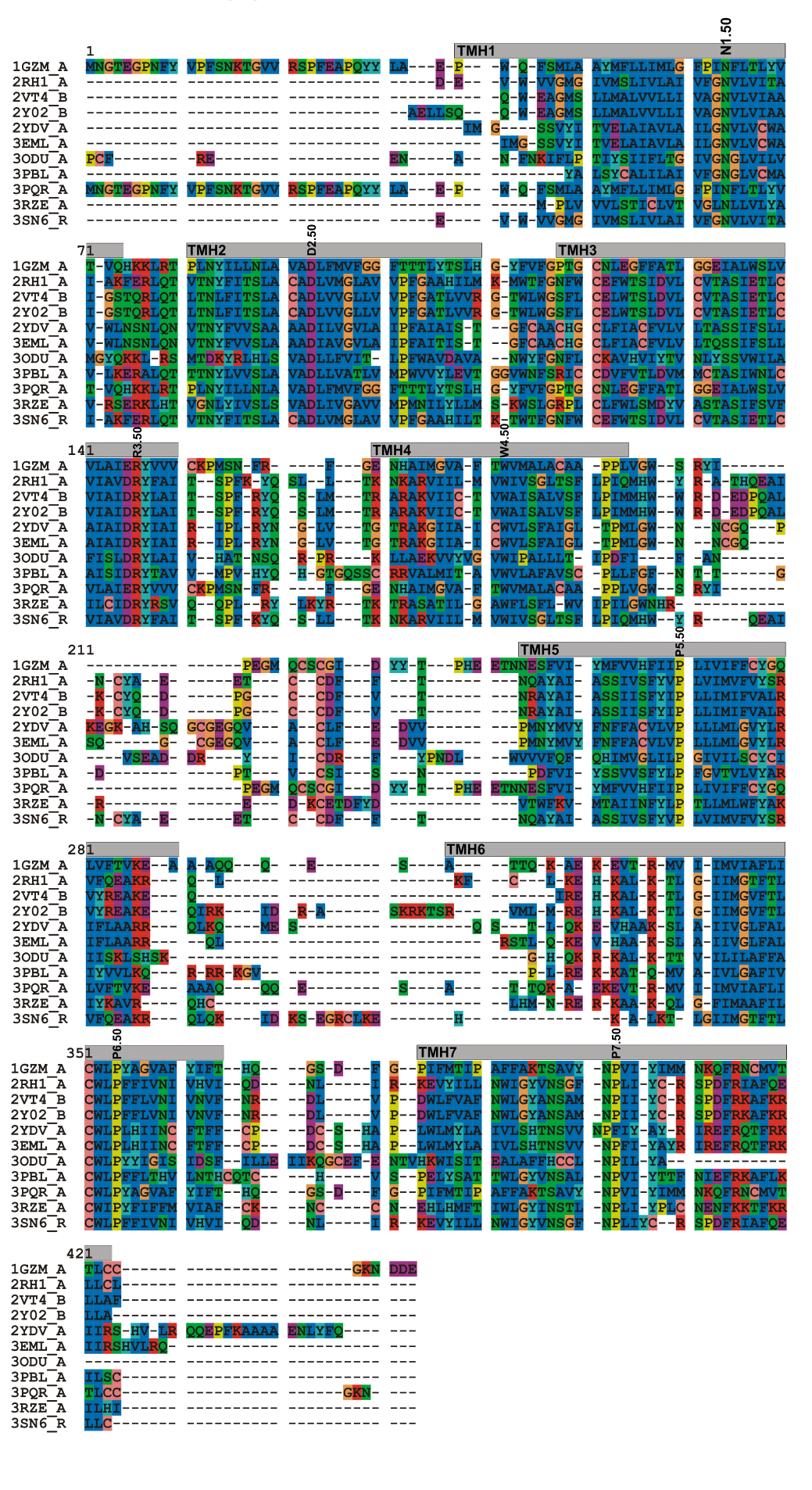


**Figure S2.** **The sequence alignment used in GPCRM modeling of A2AR.** A fragment which corresponds to the bulge in TMH4 is marked by a square box. The template with the bulge in the structure (2VT4 – β1AR) is aligned against the target sequence (A2AR) without any gaps in that fragment while the template without the bulge (3RZE – H1R) is aligned with a one-residue gap.


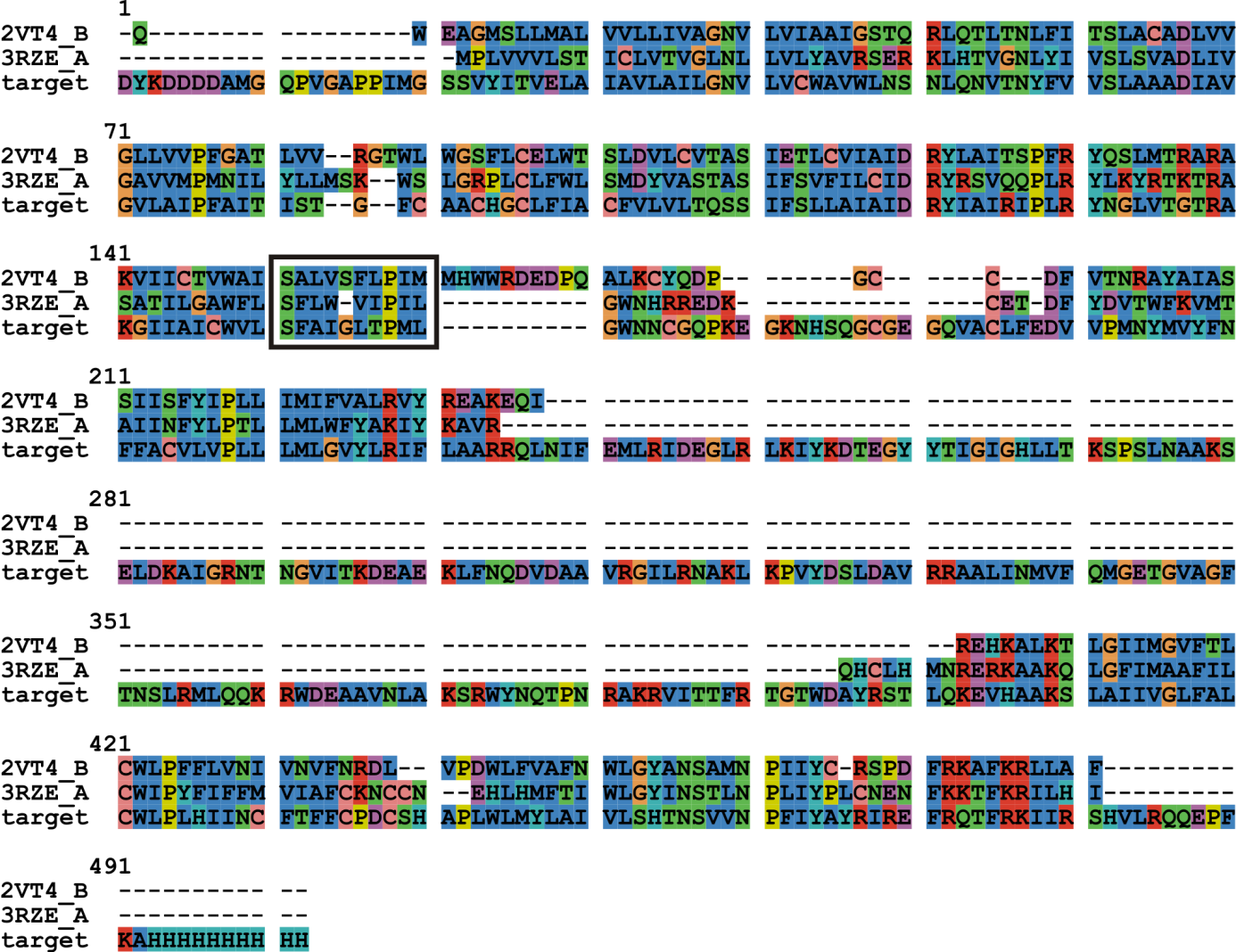


**Figure S3.** **The sequence alignment used in GPCRM modeling of κ-opioid receptor.** A fragment which corresponds to the lack of bulge in TMH2 is marked in the alignment (a square box). The template without the TMH2 bulge (3ODU – CXCR4) is aligned against the target sequence (κ-opioid receptor) without any gap in that fragment, while the template with the TMH2 (3RZE – H1R) bulge is aligned with a one-residue gap.


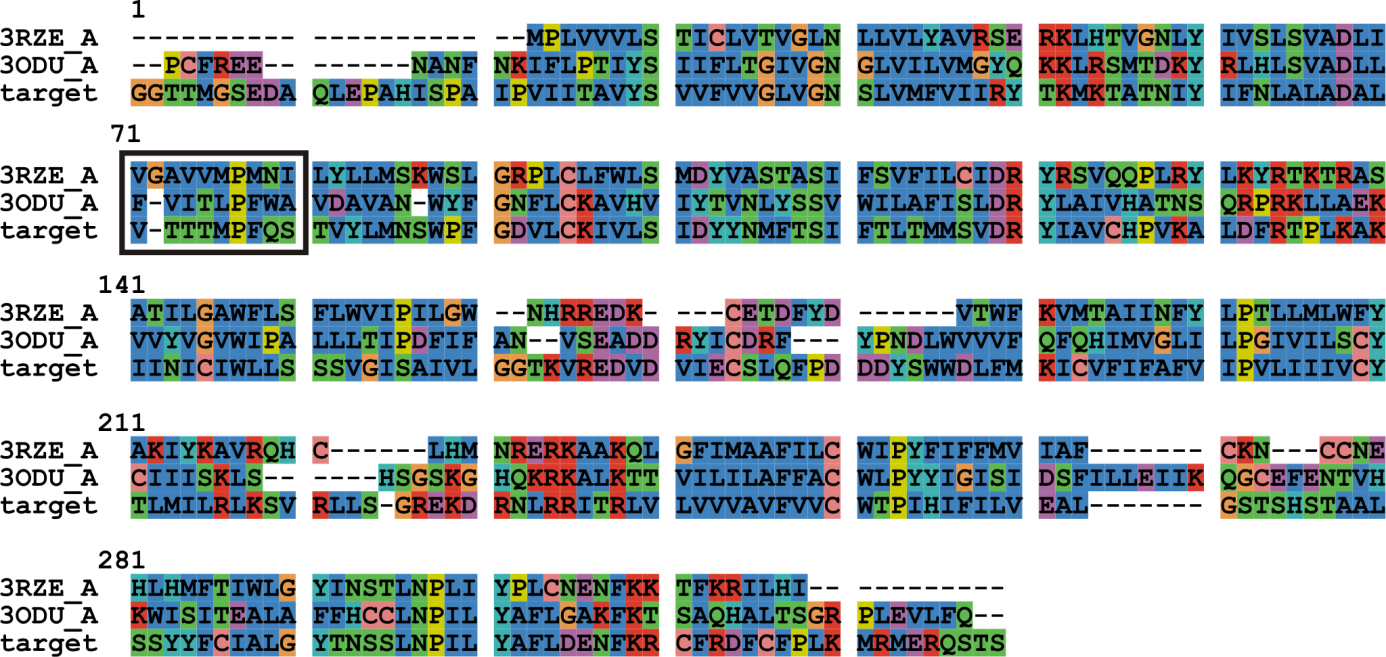


**Figure S4. The model of κ-opioid receptor (PDB id: 4DJH).** The model (green) was generated by GPCRM and superposed on the crystal structure (blue) and templates used in the model building: the histamine H1R (grey) and the CXCR4 receptor (pink). The bulge observed in TMH2 in H1R was removed and was not transferred to the κ-opioid model. Nevertheless, averaging of H1R and CXCR4 coordinates in TMH1 did not result in the proper kink of TMH1 proving limitations of the Modeller software.


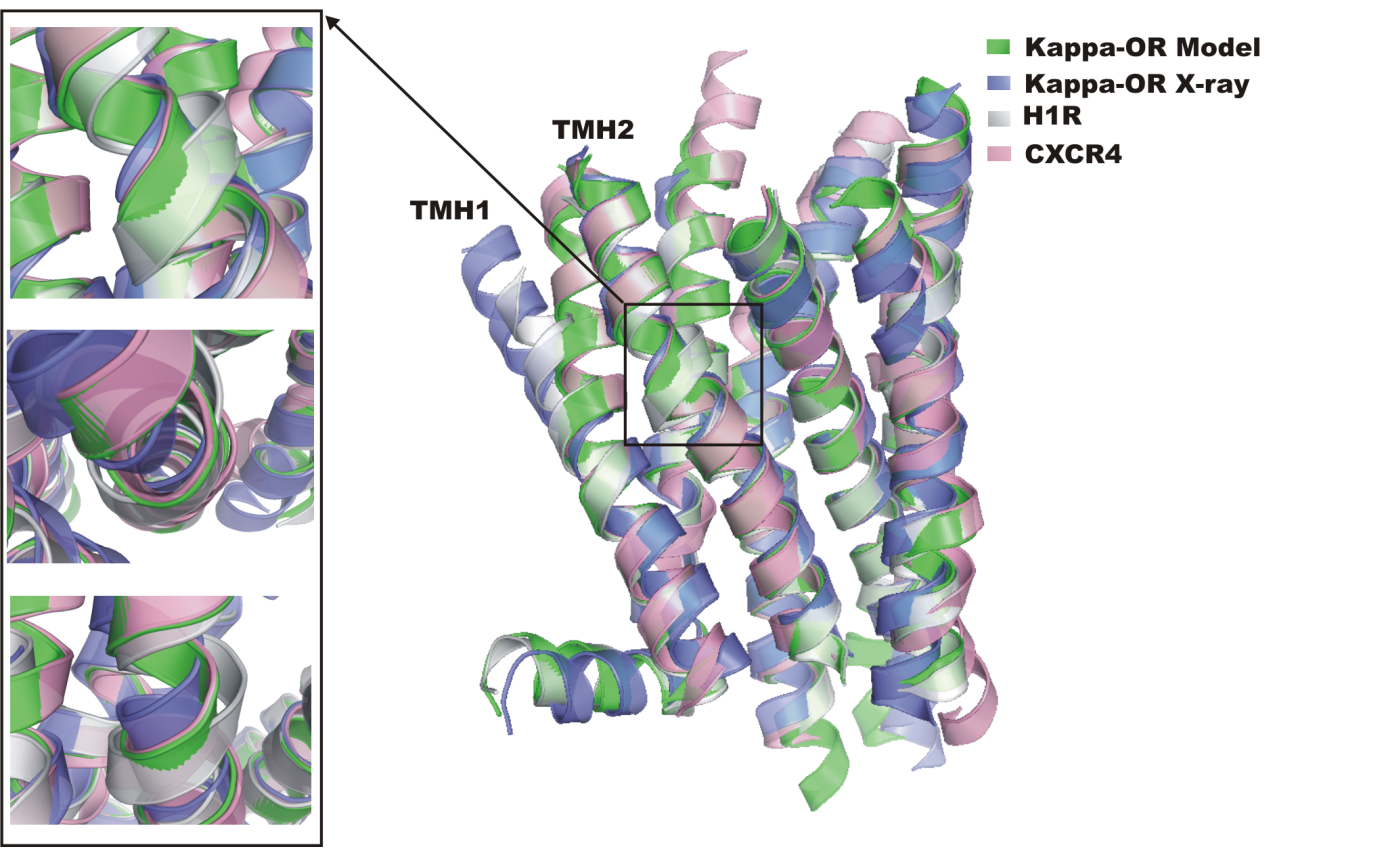


**Figure S5.** **Models of κ-opioid receptor (4DJH) generated by currently available methods.** All models are superposed on the crystal structure (blue). The bulge in TMH2 which is not present in the crystal structure is depicted. Templates used in the model building by each method are as follows: rhodopsin (ModWeb/ModBase), β1AR (GPCRDB and GPCR-Modsim), β1AR together with β2AR, A2A and rhodopsin (SSFE).


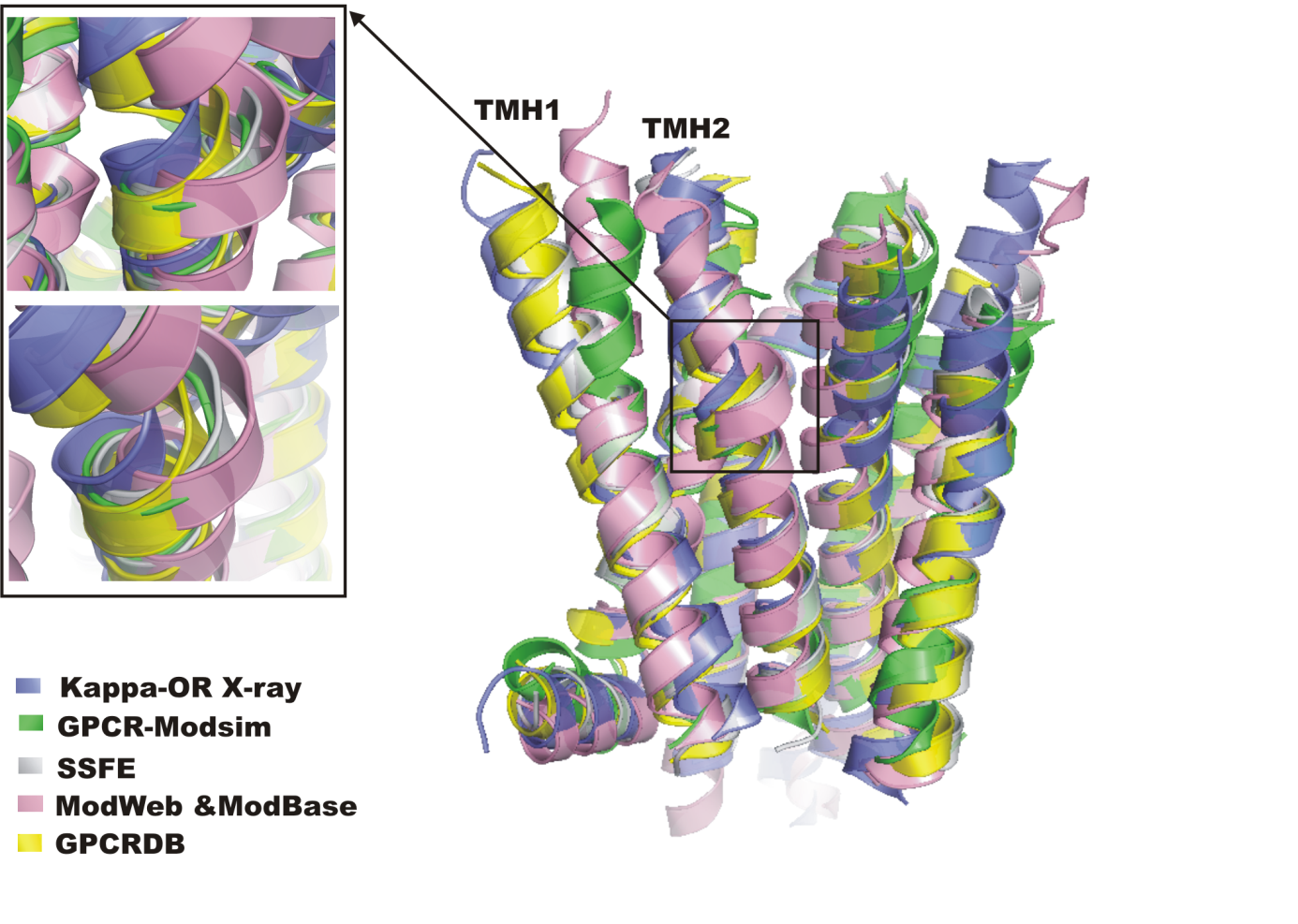


**Figure S6.** **Ligand docking to GPCRM-generated homology models versus self-docking: β2AR (A), H1R (B), CXCR4 (C) and metarhodopsin II (D).** The reference crystal complexes with indicated polar contacts (yellow dashed lines) are shown in grey, while the docked ligand poses are depicted in yellow. GPCRM-generated homology models of receptors are shown in green. Left panels show the best poses obtained from docking to corresponding protein homology models. Right panels show results of self-docking to crystal structures (PDB id: 3SN6, 3RZE, 3ODU, 3PQR). Most polar contacts were preserved except for: Ser203 (A), Thr112 (B), Asp97 (C). Although Ile189 and Tyr191 in the EC2 loop are not as deep in the binding pocket as in the crystal structure of metarhodopsin II (D), retinal was positioned in the homology model with the proper orientation of the β-ionone ring (left panel) contrary to the self-docking results (right panel).

**
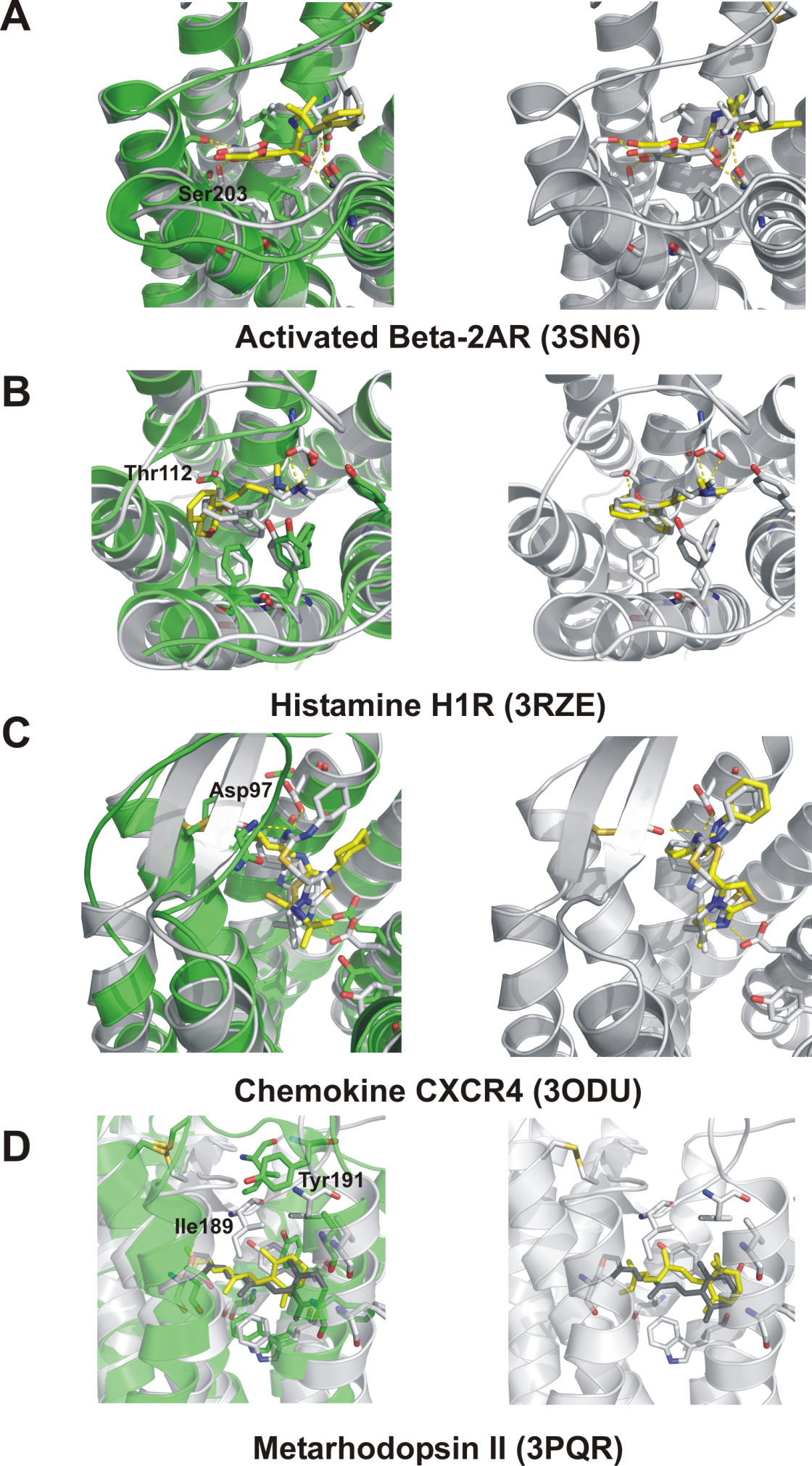
**

**Supplemental Methods**

Alignment generation

The importance of alignment accuracy cannot be overestimated in comparative modeling [[23](#_ENREF_23)]. Even in a quite straightforward alignment of GPCR sequences in which distinctive sequence motifs in TM helices help to align the whole TM region properly there are certain issues which may cause pitfalls of the whole modeling. Aligning of loop regions with a number of bridged and unbridged cysteines may require a careful inspection as they are frequently involved in the ligand binding [[24](#_ENREF_24)]. Detection of bulges and kinks in TM helices is also a challenging problem. Therefore, we implemented three methods for alignment generation: a simple pairwise sequence alignment (PSA), a multiple sequence alignment (MSA) of a target and all GPCRM templates, as well as a merging of alignments corresponding to target and template sequence profiles (a profile-profile alignment). A profile-profile alignment is computed by MUSCLE - an efficient method for multiple sequence alignments based on progressive alignments refined by tree-dependent restricted partitioning, which is much faster and more accurate (according to our tests – data not shown) than, for example, ClustalW2 [[25](#_ENREF_25)]. The importance of the sequence distribution in a profile was addressed, for example, in [[26](#_ENREF_26)]. For that reason, we provided a possibility to change the e-value range or the phylogenetic tree branch cutoff to underweight very similar sequences in the final profile.

Visual inspection revealed that none of the methods used in GPCRM was able to provide an acceptable alignment of all important functional motifs in all tested cases. Therefore, we have added a so called ‘anchored realignment’ step to GPCRM. During that step the original alignment of sequence motifs located in TMs is improved and large gaps from the regions corresponding to TM helices are removed. Following an important remark from [[2](#_ENREF_2)], concerning sequence alignments of the CXCR4 chemokine receptor with all GPCRs available in PDB, we decided to permit for one-residue gaps inside the TM helices. As we tested (data not shown) such a slight alignment manipulation did not cause disruption of a protein model generated by Modeller. Two-residue and longer gaps might cause disruption of a helix during the model building (data not shown). For that reason, such gaps are allowed only in the GPCRM advanced user mode.

All generated pairwise alignments are assessed by a similarity score, following [[27](#_ENREF_27)], and the best alignment out of three generated is selected. Here, we use the BLOSUM62 substitution matrix to compute the so-called ‘substitution scores’ and the gap extension penalty: 0.2 with a quite low gap opening penalty (2.0) for computation of 'gap scores'. A comment is needed about decreasing the importance of gap penalties in such a scoring scheme in comparison to, for example, [[28](#_ENREF_28)]. Some of the gaps in the alignment are forcedly introduced by GPCRM since they are translocated from the interior of TM helices to the outer loop regions to preserve a proper alignment of important functional motifs and the integrity of TMHs. Therefore, we reduced gap penalizing in the GPCRM pipeline as much as it was possible to preserve the sequence similarity as the main factor to assess the alignments. If more than one template is selected in the first step of the GPCRM modeling procedure, the alignments generated for every template are compared with each other to confirm that TM helices are located in the same place in the corresponding target sequence. If a significant difference in location of TM helices is reported another set of target-template alignments is selected. The process is repeated iteratively, until concurrence between alignments is reached. Further on, to proceed to the Modeller model building step GPCRM merges iteratively all selected target-template alignments (if more than one template was chosen) and detects extra and intracellular loops together with N and C-termini for the loop modeling. Such efficient reconciliation of alignments in GPCRM is inevitable since in our implementation of Modeller a GPCR model is built based on the average of template structures and not on their sum as in, e.g., SSFE.

Model building

During the model building by Modeller, the accessible protein conformational space is defined by the simplified CHARMM force field components [[29](#_ENREF_29)] and spatial restraints derived from a template (or templates) expressed as probability density functions. If more than one template is selected a protein model is built as a weighted average of given template structures depending on residue type, main-chain conformation and the local sequence similarity [[30](#_ENREF_30)]. We also used a new functionality which was recently added to Modeller: a DOPE model building and loop modeling method [[31](#_ENREF_31)]. The DOPE method is based on statistical potentials which are widely used not only in protein model quality assessment [[32](#_ENREF_32)] but also in protein folding [[33](#_ENREF_33),[34](#_ENREF_34)]. Statistical potentials (named also knowledge-based potentials) which are used in Modeller were derived mainly for globular proteins. Limited structural space of membrane proteins in PDB is a serious obstacle to derive reliable statistical potentials for them which will perform as good as in case of globular proteins. To date, there are only few studies on knowledge-based potentials derived from membrane proteins structures used, e.g., in MQAP (model quality assessment programs) [[35](#_ENREF_35)]. Following the work of [[36](#_ENREF_36)] and our own tests on the members of the GPCR family (Table 2) we decided to set the number of templates used in the modeling up to two in the automatic user mode within GPCRM. Yet, the advanced user mode enables one to incorporate even all template structures from our database. If a disulfide bridge is present in a template structure its geometry is copied directly to the target protein model. As for the Rosetta modeling step in GPCRM, disulfide bridges are included by a ‘-fix_disulf’ option.

Loop modeling

Unless the user specifies differently in the GPCRM advanced mode, the DOPE loop refinement based on pseudo-energy optimization is performed after the model building step. It is also possible to define regions of the model which should be refined by Modeller by providing the specific residue span. The only limitation in the loop modeling by Modeller is the maximal length of a loop (20 residues), as longer loops are refined by Modeller with lesser precision than short ones [[37](#_ENREF_37)]. After the Modeller step, the second loop modeling method is used for the 10 top selected models - the cyclic coordinate descent (CCD) [[38](#_ENREF_38)], which is a fragment-based algorithm implemented in Rosetta accompanied in GPCRM by the Rosetta fragments picking [[39](#_ENREF_39)] and PsiPred secondary structure predictions [[40](#_ENREF_40)]. At the Rosetta stage there is no limitation in the loop length since Rosetta assisted with the PsiPred secondary structure predictions performs much better in modeling of long loops than Modeller does, according to our studies. Moreover, modeling of long loops by Rosetta is slightly improved in GPCRM due to the usage of a 'cut-point' which divides a loop in two fragments separately refined. Such cut-points could be, for example, EC2 disulfide bridges. The loop refinement by Rosetta can be run in two modes: fast (less accurate) and slow (more precise) differing by the time of computations and the number of conformations generated. As we mentioned before, Rosetta loop modeling does not include any information about the membrane. Consequently some longer extra or intracellular loops and N or C-termini may artificially cross the membrane and fit to TMHs. To overcome such limitation, GPCRM scores Rosetta loop models by computing Z-coordinates of C-alpha atoms of residues inside the loops. The Z-coordinate, defined as a distance from a membrane center, is obtained using the implicit membrane location of the most similar template structure derived from the OPM database [[41](#_ENREF_41)]. If there are some C-alpha atoms in loops which penetrate the membrane, such protein model is discarded. Protein models which passed the Z-coordinate filter are sorted according to the all-atom Rosetta total energy score and the 10 top models are selected as the final result of the GPCRM method.

**References**

1. Lebon G, Warne T, Edwards PC, Bennett K, Langmead CJ, et al. (2011) Agonist-bound adenosine A2A receptor structures reveal common features of GPCR activation. Nature 474: 521-525.

2. Kufareva I, Rueda M, Katritch V, Stevens RC, Abagyan R (2011) Status of GPCR Modeling and Docking as Reflected by Community-wide GPCR Dock 2010 Assessment. Structure 19: 1108-1126.

3. Li J, Edwards PC, Burghammer M, Villa C, Schertler GF (2004) Structure of bovine rhodopsin in a trigonal crystal form. Journal of molecular biology 343: 1409-1438.

4. Okada T, Sugihara M, Bondar AN, Elstner M, Entel P, et al. (2004) The retinal conformation and its environment in rhodopsin in light of a new 2.2 A crystal structure. Journal of molecular biology 342: 571-583.

5. Cherezov V, Rosenbaum DM, Hanson MA, Rasmussen SG, Thian FS, et al. (2007) High-resolution crystal structure of an engineered human beta2-adrenergic G protein-coupled receptor. Science 318: 1258-1265.

6. Warne T, Serrano-Vega MJ, Baker JG, Moukhametzianov R, Edwards PC, et al. (2008) Structure of a beta1-adrenergic G-protein-coupled receptor. Nature 454: 486-491.

7. Warne T, Moukhametzianov R, Baker JG, Nehme R, Edwards PC, et al. (2011) The structural basis for agonist and partial agonist action on a beta(1)-adrenergic receptor. Nature 469: 241-244.

8. Jaakola VP, Griffith MT, Hanson MA, Cherezov V, Chien EY, et al. (2008) The 2.6 angstrom crystal structure of a human A2A adenosine receptor bound to an antagonist. Science 322: 1211-1217.

9. Wu B, Chien EY, Mol CD, Fenalti G, Liu W, et al. (2010) Structures of the CXCR4 chemokine GPCR with small-molecule and cyclic peptide antagonists. Science 330: 1066-1071.

10. Chien EY, Liu W, Zhao Q, Katritch V, Han GW, et al. (2010) Structure of the human dopamine D3 receptor in complex with a D2/D3 selective antagonist. Science 330: 1091-1095.

11. Choe HW, Kim YJ, Park JH, Morizumi T, Pai EF, et al. (2011) Crystal structure of metarhodopsin II. Nature 471: 651-655.

12. Xu F, Wu H, Katritch V, Han GW, Jacobson KA, et al. (2011) Structure of an agonist-bound human A2A adenosine receptor. Science 332: 322-327.

13. Shimamura T, Shiroishi M, Weyand S, Tsujimoto H, Winter G, et al. (2011) Structure of the human histamine H1 receptor complex with doxepin. Nature 475: 65-70.

14. Rasmussen SG, DeVree BT, Zou Y, Kruse AC, Chung KY, et al. (2011) Crystal structure of the beta2 adrenergic receptor-Gs protein complex. Nature 477: 549-555.

15. Kruse AC, Hu J, Pan AC, Arlow DH, Rosenbaum DM, et al. (2012) Structure and dynamics of the M3 muscarinic acetylcholine receptor. Nature 482: 552-556.

16. Wu H, Wacker D, Mileni M, Katritch V, Han GW, et al. (2012) Structure of the human kappa-opioid receptor in complex with JDTic. Nature 485: 327-332.

17. Manglik A, Kruse AC, Kobilka TS, Thian FS, Mathiesen JM, et al. (2012) Crystal structure of the micro-opioid receptor bound to a morphinan antagonist. Nature 485: 321-326.

18. Granier S, Manglik A, Kruse AC, Kobilka TS, Thian FS, et al. (2012) Structure of the delta-opioid receptor bound to naltrindole. Nature 485: 400-404.

19. Haga K, Kruse AC, Asada H, Yurugi-Kobayashi T, Shiroishi M, et al. (2012) Structure of the human M2 muscarinic acetylcholine receptor bound to an antagonist. Nature 482: 547-551.

20. Hanson MA, Roth CB, Jo E, Griffith MT, Scott FL, et al. (2012) Crystal structure of a lipid G protein-coupled receptor. Science 335: 851-855.

21. Thompson AA, Liu W, Chun E, Katritch V, Wu H, et al. (2012) Structure of the nociceptin/orphanin FQ receptor in complex with a peptide mimetic. Nature 485: 395-399.

22. Roberts E, Eargle J, Wright D, Luthey-Schulten Z (2006) MultiSeq: unifying sequence and structure data for evolutionary analysis. BMC bioinformatics 7: 382.

23. Schwarzenbacher R, Godzik A, Grzechnik SK, Jaroszewski L (2004) The importance of alignment accuracy for molecular replacement. Acta crystallographica Section D, Biological crystallography 60: 1229-1236.

24. Wheatley M, Wootten D, Conner MT, Simms J, Kendrick R, et al. (2012) Lifting the lid on GPCRs: the role of extracellular loops. British journal of pharmacology 165: 1688-1703.

25. Edgar RC (2004) MUSCLE: multiple sequence alignment with high accuracy and high throughput. Nucleic acids research 32: 1792-1797.

26. Poleksic A, Fienup M (2008) Optimizing the size of the sequence profiles to increase the accuracy of protein sequence alignments generated by profile-profile algorithms. Bioinformatics 24: 1145-1153.

27. Ye X, Wang G, Altschul SF (2011) An assessment of substitution scores for protein profile-profile comparison. Bioinformatics 27: 3356-3363.

28. Pirovano W, Feenstra KA, Heringa J (2008) PRALINETM: a strategy for improved multiple alignment of transmembrane proteins. Bioinformatics 24: 492-497.

29. Eswar N, Webb B, Marti-Renom MA, Madhusudhan MS, Eramian D, et al. (2006) Comparative protein structure modeling using Modeller. Current protocols in bioinformatics / editoral board, Andreas D Baxevanis [et al] Chapter 5: Unit 5 6.

30. Sali A, Blundell TL (1993) Comparative protein modelling by satisfaction of spatial restraints. Journal of molecular biology 234: 779-815.

31. Shen MY, Sali A (2006) Statistical potential for assessment and prediction of protein structures. Protein Science 15: 2507-2524.

32. Sippl MJ (1993) Recognition of errors in three-dimensional structures of proteins. Proteins 17: 355-362.

33. Kolinski A (2004) Protein modeling and structure prediction with a reduced representation. Acta biochimica Polonica 51: 349-371.

34. Latek D, Kolinski A (2008) Contact prediction in protein modeling: scoring, folding and refinement of coarse-grained models. BMC Structural Biology 8: 36.

35. Ray A, Lindahl E, Wallner B (2010) Model quality assessment for membrane proteins. Bioinformatics 26: 3067-3074.

36. Larsson P, Wallner B, Lindahl E, Elofsson A (2008) Using multiple templates to improve quality of homology models in automated homology modeling. Protein science : a publication of the Protein Society 17: 990-1002.

37. Jamroz M, Kolinski A (2010) Modeling of loops in proteins: a multi-method approach. BMC Structural Biology 10: 5.

38. Canutescu AA, Dunbrack RL, Jr. (2003) Cyclic coordinate descent: A robotics algorithm for protein loop closure. Protein Science 12: 963-972.

39. Gront D, Kulp DW, Vernon RM, Strauss CE, Baker D (2011) Generalized fragment picking in Rosetta: design, protocols and applications. PloS one 6: e23294.

40. McGuffin LJ, Bryson K, Jones DT (2000) The PSIPRED protein structure prediction server. Bioinformatics 16: 404-405.

41. Lomize MA, Pogozheva ID, Joo H, Mosberg HI, Lomize AL (2012) OPM database and PPM web server: resources for positioning of proteins in membranes. Nucleic Acids Research 40: D370-376.
